# Chinese elderly migrants' loneliness, anxiety and depressive symptoms: The mediation effect of perceived stress and resilience

**DOI:** 10.3389/fpubh.2022.998532

**Published:** 2022-08-25

**Authors:** Hao Wang, Yanjie Hou, Lin Zhang, Man Yang, Ruyue Deng, Jun Yao

**Affiliations:** ^1^School of Nursing, Nanjing Medical University, Nanjing, China; ^2^School of Health Policy and Management, Nanjing Medical University, Nanjing, China; ^3^Institute of Healthy Jiangsu Development, Nanjing Medical University, Nanjing, China

**Keywords:** loneliness, anxiety, depression, elderly migrants, mediation analysis

## Abstract

Elderly migrants who face the dual challenges of aging and migration are more likely to suffer psychological disorders. Existing research has demonstrated a link between loneliness and psychological disorders in the general elderly population. However, we know little about the relationships among elderly migrants, and the psychological mechanisms linking them. This study aims to examine the effects of loneliness on anxiety and depressive symptoms among Chinese elderly migrants, and explore the mediating roles of perceived stress and resilience. All 654 participants were recruited in Nanjing, Jiangsu Province, China. Results showed that loneliness was significantly correlated with anxiety and depressive symptoms (*p* < 0.001). Perceived stress and resilience mediated the relationship between loneliness and anxiety symptoms. The mediating effect of perceived stress was 0.128 (Bootstrap 95% CI: 0.092–0.168, Ratio = 37.4%). Resilience was 0.026 (Bootstrap 95% CI: 0.005–0.049, Ratio = 7.6%). Furthermore, perceived stress and resilience also mediated the relationship between loneliness and depressive symptoms. The mediating effects were 0.111 and 0.043, respectively (Bootstrap 95% CI: 0.073–0.151, Ratio = 27.9%; Bootstrap 95% CI: 0.020–0.069, Ratio = 10.8%). All the mediating effects were significant because the bootstrap 95% CIs did not contain zero. Overall, our findings suggested that loneliness not only can directly influence elderly migrants' anxiety and depressive symptoms but also by increasing perceived stress or decreasing resilience.

## Introduction

Population migration is a key issue and a significant factor in the sustainable population development of China (1). Population migration is a complex process involving multiple changes, which can bring about a series of problems such as lack of social networks and social support, language and cultural differences, and discrimination, all of them may have a negative effect on migrants' mental health (2, 3). According to “Report on China's Migrant Population Development”, during the 15 years from 2000 to 2015, the number of elderly migrants increased from 5.03 to 13.04 million, with a 6.3% annual increase (1). With the rapid increase of elderly migrants, more and more researchers have begun to pay attention to their physical and mental health.

Anxiety and depression are two common psychological disorders in later life (4–7), and are significantly associated with low quality of life and high risk of suicide (8–11). The West China Health and Aging Trend Study shows that 20.8% elderly people have the anxiety symptoms, and 19.6% have depressive symptoms (7). Over the past few decades, researchers have carried out numerous studies on the factors that may put people at risk of anxiety and depression. Previous studies have confirmed that loneliness is a key predictor of anxiety and depressive symptoms (6, 12). However, researchers did not deliberately distinguish elderly migrants from non-migrants, although previous studies have shown that migrants are more likely to suffer psychological disorders than non-migrants (13–15). And little is known the psychological mechanisms linking them. So, we conducted this study aimed at examining the effects of loneliness on anxiety and depressive symptoms among Chinese elderly migrants, and attempted to explore the mediating roles of perceived stress and resilience.

### Loneliness, anxiety and depression

Loneliness refers to the subjective feeling of inadequate social relations (16) and has become a public health problem (17). A national survey including 20,255 Chinese elderly people aged 60 and above showed that 29.6% participants reported “often felt lonely” (18). For elderly people, they will face a shrinking of social network, decrease of social interaction, and loss of social roles as they age, all of them may make them feel lonely (6). An overview of 40 systematic reviews found that loneliness is associated with the increased mortality and negative mental health outcomes (19).

Anxiety and depression are common mental health problems in the elderly and are the hot topics of gerontological research. Numeral studies have confirmed that loneliness is associated with anxiety and depressive symptoms in the elderly (6, 12). Creese et al. (20) found that loneliness was a risk factor for anxiety and depression in the elderly both before and during COVID-19 (20). In addition, a 5-year longitudinal study conducted in Chicago found that loneliness can predict the subsequent increase of depressive symptoms in the elderly (21).

### The mediating role of perceived stress and resilience

Stress is an important risk factor for physical and mental health, and can affect health not only directly through autonomic and neuroendocrine responses, but also indirectly through changes in health-related behaviors (22). Perceived stress is the assessment of the level of threat from the stressor they face (23, 24). In past studies, researchers have found that people with high levels of perceived stress have high risks to suffer anxiety and depression (21). Loneliness is a painful experience, and has been confirmed as a stressor (25, 26). Burke and Segrin (25) found that loneliness had a positive effect on perceived stress. Similarly, Cacioppo et al. (27) found that people who felt lonely had a higher level of perceived stress than non-lonely people, even when they were exposed to a similar frequency and intensity of stressor, and even when they were relaxed. Moreover, several studies in Chinese elderly population confirmed that perceived stress was a crucial mediator between the relationship of loneliness and mental health (28–30). So, we speculate that perceived stress plays a crucial role between the relationships of loneliness on anxiety and depressive symptoms among Chinese elderly migrants.

Resilience refers to the ability of a person to adapt to and recover from trauma, adversity and stressor (31, 32). It is well-known that resilience is a protective factor for health and wellbeing, and is a key contributor of successful aging (33–35). Several empirical studies have confirmed that people with high resilience are at a low risk to suffer anxiety and depression (6, 36). Kumpfer's resilience framework suggests that resilience can mediate the relationships between adversity and its outcomes and propel a person to grow by facing adversity (37). A study in nursing homes found that loneliness had a negative effect on elderly people' depression and resilience played a mediating role between this association (38). Moreover, researchers argued that resilience can be influenced by internal factors (biological and psychological) and external factors (environmental) (34, 39). For the elderly migrants, the environmental changes coming with migration may influence their resilience (40). So, we think that elderly migrants' resilience is worth investigating and it may play a crucial role between the relationship of loneliness on anxiety and depressive symptoms among Chinese elderly migrants.

### The current study

**Figure 1:**
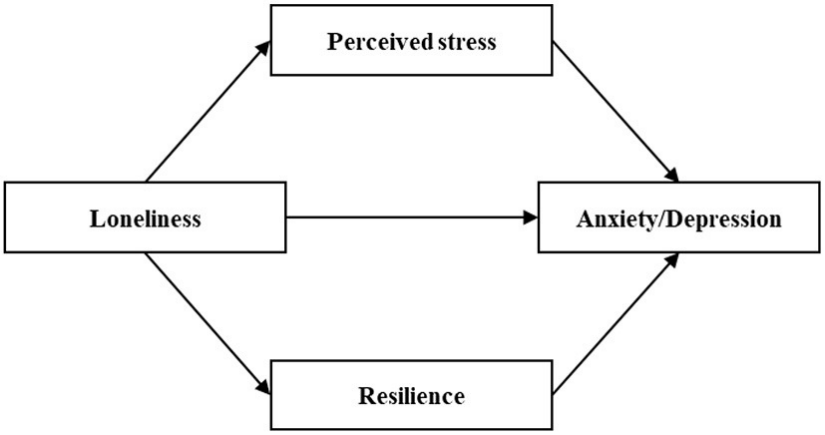
Hypothesized relationships between loneliness, perceived stress, resilience, anxiety and depression.

From what has been discussed above, we can find that there exists a significant reciprocal relationship between loneliness, perceived stress, resilience, anxiety and depressive symptoms, and perceived stress and resilience maybe play crucial roles between the relationships of loneliness on anxiety and depressive symptoms. Elderly migrants who face the dual challenges of aging and migration are more likely to suffer psychological disorders. However, most of the previous studies on the effects of loneliness on anxiety and depression did not distinguish elderly migrants from non-migrants. The purpose of this study is to examine the relationships between loneliness on anxiety and depressive symptoms among Chinese elderly migrants, and explore the mediating roles of perceived stress and resilience. Thus, we propose the following hypotheses (Figure 1).

Hypothesis 1. Loneliness is a positive predictor of anxiety and depressive symptoms among Chinese elderly migrants.

Hypothesis 2. Perceived stress plays a mediating role in the relationship between loneliness and anxiety symptom among Chinese elderly migrants.

Hypothesis 3. Perceived stress plays a mediating role in the relationship between loneliness and depressive symptom among Chinese elderly migrants.

Hypothesis 4. Resilience plays a mediating role in the relationship between loneliness and anxiety symptom among Chinese elderly migrants.

Hypothesis 5. Resilience plays a mediating role in the relationship between loneliness and depressive symptom among Chinese elderly migrants.

## Methods

### Participants

The data in this study came from the National Social Science Foundation Project of China “A follow-up study on the mechanism of intergenerational relationship on the mental health of elderly migrants”. This project was performed from September 2019 to September 2020 in Nanjing, Jiangsu Province, China. The project first randomly selected seven districts in Nanjing (Qinhuai, Qixia, Gulou, Xuanwu, Jianye, Yuhuatai, and Jiangning District), then randomly selected three communities in each district, and finally recruited elderly migrants who met the inclusion criteria in these 21 communities. All participants were informed of the purpose of the study and volunteered to participate. All participants were face-to-face interviewed using a structured questionnaire. All interviewers had medical research background and received uniform and standardized training prior to the project. This study used the first phase survey data of the project. Inclusion criteria were: (1) aged 60 and above; (2) household registration not moved to Nanjing; (3) moved to Nanjing ≤ 10 years. A total of 654 participants were included in this study after screening. The mean number of years that participants moved to Nanjing was 3.96 ± 1.96. The necessary permission to conduct this study was obtained from the ethics committee of the university.

### Measures

#### Loneliness

The UCLA 3-item loneliness scale was used to measure loneliness of elderly migrants (41). Each item is scored on a scale from 1 (hardly ever or never) to 3 (almost always) and the total score ranges from 3 to 9. Higher scores indicate higher feeling of loneliness. The Chinese version has high reliability and validity in the elderly (42, 43). Cronbach's α for the present sample was 0.866.

#### Perceived stress

Perceived Stress Scale (PSS) was used to measure perceived stress of elderly migrants over the past month (23). PSS consists of 14 items and two subscales: sense of uncontrollable and sense of nervous. Items 4, 5, 6, 7, 9, 10, and 13 belong to the uncontrollable dimension and are scored in reverse. Items 1, 2, 3, 8, 11, 12, and 14 belong to the nervous dimension. Each item is scored on a scale from 0 (never) to 4 (always) and the total score ranges from 0 to 56. Higher scores indicate higher perceived stress. The Chinese version is translated by Yang and Huang (44), and has high reliability and validity (44, 45). Cronbach's α for the present sample was 0.809.

#### Resilience

The 10-item Connor–Davidson Resilience Scale (CD-RISC-10) was used to measure resilience of elderly migrants (46). CD-RISC-10 contains 10 items. Each item is scored on a scale from 1 (never) to 5 (always) and the total score ranges from 10 to 50. Higher scores indicate higher resilience. Several studies have shown that CD-RISC-10 has high reliability and validity in the Chinese population (47, 48). Cronbach's α for the present sample was 0.922.

#### Anxiety

The Hospital Anxiety and Depression Scale-Anxiety (HADS-A) was used to measure anxiety of elderly migrants (49). HADS-A is a screening scale for anxiety which consists of seven items. Each item is scored on a scale from 0 to 3 and the total score ranges from 0 to 21. Higher scores indicate higher risks to suffer anxiety. Several studies have shown that HADS-A also has a high reliability and validity in the general population (5, 50). Cronbach's α for the present sample was 0.787.

#### Depression

The 9-item Patient Health Questionnaire (PHQ-9) was used to measure depression of elderly migrants over the past 2 weeks (51). PHQ-9 is a screening scale for depression which consists of 9 items. Each item is scored on a scale from 0 (never) to 3 (almost every day) and the total score ranges from 0 to 27. Higher scores indicate higher risks to suffer depression. The Chinese version has been widely used and demonstrated a high reliability and validity among elderly people (52, 53). Cronbach's α for the present sample was 0.806.

#### Demographic variables

Demographic data such as age, sex, marital status, education level, religious belief, household registration, retirement pension and yearly income were collected. Marital status was categorized into: divorced or widowed, married and having a spouse. Education level was classified as primary school or lower, junior or senior high school, and college or higher. Yearly income was categorized as: 0–5,000¥, 5,001–10,000¥, 10,001–20,000¥, and >20,000¥.

### Analytic strategies

Scale items were presented in Table 1. All analyses were conducted in the SPSS 26.0 software and the significance level was set at 0.05 (two-tailed). Firstly, we implemented descriptive analysis to describe the demographic characteristics of the participants. Then, we conducted the Pearson correlation analysis to examine the bivariate correlations of loneliness, perceived stress, resilience, anxiety and depression. Finally, we used the SPSS PROCESS macro 4.0 (54) to explore the mediating role of perceived stress and resilience between the relationship of loneliness and psychological distress. PROCESS Model 4 was used to build the multiple mediation models with a bootstrap sample of 5,000. The mediating effect was significant if the bootstrap 95%CI did not contain zero.

**Table 1:** Scale items of UCAL-3, PSS and CD-RISC-10.

**Scale**	**Items**
UCAL-3	How often do you feel the lack of company?
	How often do you feel that life is boring?
	How often do you feel isolated from others?
PSS	Feeling distracted by something that cannot be expected to happen.
	Feeling unable to control the important things in your life.
	Feeling jittery and stressed.
	Successfully deal with annoying life troubles.
	Feel that you can effectively deal with important changes in your life.
	Feel confident in being able to handle your own personal issues.
	Feel that things are going well.
	Find yourself unable to handle all the things you have to do.
	There are ways to control the annoying things in your life.
	Feel like you are in charge of things.
	You are often angry because many things are happening beyond your control.
	You often feel that there are things you have to accomplish.
	Able to master the way of time arrangement.
	Feel that difficult things are piling up and you can't get over them.
CD-RISC-10	I can adapt to changes.
	I can handle anything.
	I can find the humorous side of things.
	Dealing with stress makes me stronger.
	I can bounce back after illness and hardship.
	Even with the obstacles, I can still achieve my goals.
	Under pressure, I can still focus and think clearly.
	I don't get discouraged easily by failure.
	I consider myself a strong person.
	I can deal with unpleasant feelings.

## Results

### Demographic characteristics

The demographic characteristics of all 654 participants were shown in Table 2. In this study, the mean age of elderly migrants was 66.05 years old (SD = 4.67; Range = 60–86), 216 (33.0%) were males and 438 (67.0%) were females. One hundred and one (15.4%) elderly migrants had a terrible marital status (divorced or windowed). Three hundred and fifty-four (54.1%) had a low level of education (primary school or lower). Ninety-nine (15.1%) had a religious belief. Four hundred and fifty-three (69.3%) had a rural household registration. Four hundred and three (61.6%) had a retirement pension.

**Table 2:** Demographic characteristics of 654 participants.

**Variables**	**Category**	***N***	**Mean ±SD/percentage**
Age		654	66.05 ± 4.67
Gender	Male	216	33.0%
	Female	438	67.0%
Marital status	Divorced or widowed	101	15.4%
	Married with spouse	553	84.6%
Education level	Primary school or lower	354	54.1%
	Junior or senior high school	262	40.1%
	College or higher	38	5.8%
Religious belief	No	555	84.9%
	Yes	99	15.1%
Household registration	Rural	453	69.3%
	Town	201	30.7%
Retirement pension	No	251	38.4%
	Yes	403	61.6%
Yearly income (¥)	0–5,000	249	38.0%
	5,001–10,000	126	19.3%
	10,001–20,000	87	13.3%
	>20,000	192	29.4%

### Bivariate correlations of the key variables

The bivariate correlations of the key variables were presented in Table 3. The results showed that loneliness, perceived stress, resilience and anxiety were significantly correlated with each other (*p* < 0.001). As hypothesized, loneliness was positively correlated with perceived stress (*r* = 0.398, *p* < 0.001) and negatively correlated with resilience (*r* = −0.298, *p* < 0.001). In addition, loneliness was positively correlated with anxiety (*r* = 0.361, *p* < 0.001) and depression (*r* = 0.430, *p* < 0.001). Perceived stress was negatively correlated with resilience (*r* = −0.607, *p* < 0.001), and positively correlated with anxiety (*r* = 0.499, *p* < 0.001) and depression (*r* = 0.517, *p* < 0.001). Resilience was negatively correlated with anxiety (*r* = −0.378, *p* < 0.001) and depression (*r* = −0.444, *p* < 0.001). Based on the bivariate correlations between variables, we further conducted the multiple mediation analyses to explore the mediating roles of perceived stress and resilience in the following section.

**Table 3:** The results of Pearson correlation analysis.

**Variables**	**Mean**	**SD**	**1**	**2**	**3**	**4**	**5**
1. Loneliness	4.02	1.43	1				
2. Perceived stress	20.87	7.66	0.398^***^	1			
3. Resilience	33.06	7.53	−0.298^***^	−0.607^***^	1		
4. Anxiety	11.20	3.36	0.361^***^	0.499^***^	−0.378^***^	1	
5. Depression	5.00	3.99	0.430^***^	0.517^***^	−0.444^***^	0.521^***^	1

*** p < 0.001 (two-tailed).

### Mediation analyses

**Figure 2:**
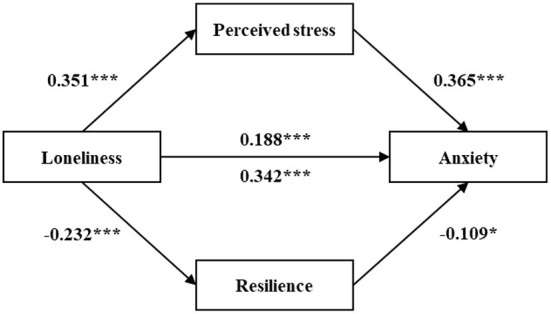
The multiple mediation model of the association between loneliness and **anxiety** through perceived stress and resilience. *p < 0.05, ***p < 0.001 (two-tailed). All values have been standardized.

**Figure 3:**
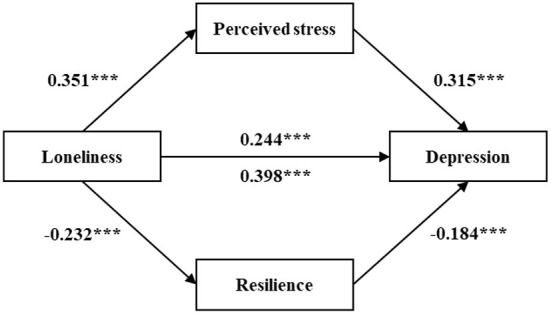
The multiple mediation model of the association between loneliness and **depression** through perceived stress and resilience. ***p < 0.001 (two-tailed). All values have been standardized.

The results of the multiple mediation analyses were shown in Tables 4, 5 and Figures 2, 3. In the multiple mediation model, loneliness was entered as the independent variable; anxiety and depression as the dependent variables; perceived stress and resilience as the mediating variables, and age, gender, marital status, education level, religious belief, household registration, retirement pension and yearly income as the control variables.

**Table 4:** The results of the multiple mediation model.

**Path**	**Effect**	**Boot SE**	**Boot LLCI**	**Boot ULCI**	**Ratio**
Total indirect effect	0.154^a^	0.020	0.116	0.195	45.0%
Indirect effect 1 (X → M1 → Y)	0.128^a^	0.020	0.092	0.168	37.4%
Indirect effect 2 (X → M2 → Y)	0.026^a^	0.011	0.005	0.049	7.6%
Compare 1 (Indirect effect 1 minus 2)	0.103^a^	0.025	0.055	0.153	

X, Loneliness; **Y, Anxiety**; M1, Perceived stress; M2, Resilience. Ratio, the ratio of the indirect effect to the total effect.

a The bootstrap 95% CIs not contain zero. Age, sex, marital status, education level, religious belief, household registration, retirement pension and yearly income were analyzed as control variables. All values have been standardized.

**Table 5:** The results of the multiple mediation model.

**Path**	**Effect**	**Boot SE**	**Boot LLCI**	**Boot ULCI**	**Ratio**
Total indirect effect	0.154^a^	0.020	0.115	0.194	38.7%
Indirect effect 1 (X → M1 → Y)	0.111^a^	0.020	0.073	0.151	27.9%
Indirect effect 2 (X → M2 → Y)	0.043^a^	0.012	0.020	0.069	10.8%
Compare 1 (Indirect effect 1 minus 2)	0.068^a^	0.026	0.016	0.120	

X, Loneliness; **Y, Depression**; M1, Perceived stress; M2, Resilience. Ratio, the ratio of the indirect effect to the total effect.

a The bootstrap 95% CIs not contain zero. Age, sex, marital status, education level, religious belief, household registration, retirement pension and yearly income were analyzed as control variables. All values have been standardized.

Table 4 and Figure 2 showed the mediating role of perceived stress and resilience in the relationship between loneliness and anxiety. The total effect of loneliness on anxiety was 0.342 (*p* < 0.001). When perceived stress and resilience entered as mediators, the effect decreased (β = 0.188, *p* < 0.001). The total indirect effect was 0.154 (95% CI: 0.116, 0.195) and the ratio to the total effect was 45.0% (0.154/0.342). The indirect effect of perceived stress was 0.128 (95% CI: 0.092, 0.168; Ratio = 37.4%). Resilience was 0.026 (95% CI: 0.005, 0.049; Ratio = 7.6%). All the indirect effects were significant because the bootstrap 95% CIs did not contain zero. Hypothesis 1, hypothesis 2 and hypothesis 4 were confirmed. In addition, the indirect effect of perceived stress was significantly higher than resilience, as the bootstrap 95% CIs did not contain zero (95% CI: 0.055, 0.153).

Table 5 and Figure 3 showed the mediating role of perceived stress and resilience in the relationship between loneliness and depression. The total effect of loneliness on depression was 0.398 (*p* < 0.001). When perceived stress and resilience entered as mediators, the effect decreased (β = 0.244, *p* < 0.001). The total indirect effect was 0.154 (95% CI: 0.115, 0.194; Ratio = 38.7%). The indirect effect of perceived stress was 0.111 (95% CI: 0.073, 0.151; Ratio = 27.9%). Resilience was 0.043 (95% CI: 0.020, 0.069; Ratio = 10.8%). All the indirect effects were significant because the bootstrap 95% CIs did not contain zero. Hypothesis 1, hypothesis 3 and hypothesis 5 were confirmed. In addition, the indirect effect of perceived stress was significantly higher than resilience, as the bootstrap 95% CIs did not contain zero (95% CI: 0.016, 0.120).

## Discussion

The main purpose of this study is to examine the relationships between loneliness, anxiety and depression among Chinese elderly migrants, and explore the psychological mechanisms linking them. In line with our expectations, all hypotheses we presented before were confirmed. Through the Pearson correlation analysis, we found that there was a significant pairwise correlation between loneliness, perceived stress, resilience, anxiety and depression. Based on these correlations, we further conducted the multiple mediation analyses. One major finding of the multiple mediation analysis is that loneliness is a positive predictor of anxiety and depression among Chinese elderly migrants, meaning that elderly migrants with higher loneliness have greater anxiety and depression. Another one is that elderly migrants' reported status of perceived stress and resilience strongly mediated the relationship of loneliness on anxiety and depression. The total mediating effects of perceived stress and resilience are 45.0 and 38.7%, respectively, revealing that our mediating variables play crucial roles in explaining the relationships of loneliness on anxiety and depression. In short, loneliness can not only directly affect anxiety and depression, but also can indirectly affect them by perceived stress or resilience.

### The direct effect of loneliness on anxiety and depression

In line with hypothesis 1, loneliness is a positive predictor of elderly migrants' anxiety and depression. Higher loneliness is associated with greater anxiety and depressive symptoms. The result is consistent with those in the general elderly population (6, 20). However, the scores of loneliness, anxiety and depression are lower than previous studies in the general elderly population, which are contrary to what we envisioned. This phenomenon may be explained by the multidimensional properties of loneliness and the characteristics of Chinese elderly migrants. Loneliness is regarded as a multidimensional concept that includes social loneliness and emotional loneliness, the former being influenced by social networks, and the latter by intimate relationships (55, 56). Previous studies have found that both of social loneliness and emotional loneliness are significant associated with psychological health (57, 58). For the elderly migrants, they usually face a shrinking of social network and difficulties of social integration due to the change of environment as well as language and cultural differences that come with migration (3, 59, 60). In addition, due to the fact that the main purpose of migration among Chinese elderly migrants is providing care for their grandchildren, the average age of the samples in the study of elderly migrants is significantly lower than in the study of the general elderly population (66.05 ± 4.67 in this study) and the intimate relationship may be improved. We think that all of these lead to the difference between our study and studies in the general elderly population.

### The mediating role of perceived stress and resilience

In line with our hypotheses, perceived stress plays a mediating role between the relationship of loneliness on anxiety and depression. The mediating effects of perceived stress are 37.4 and 27.9%, respectively. This finding is consistent with previous studies revealing that loneliness is a risk factor for anxiety and depression, and is associated with high levels of perceived stress (28, 61). Previous studies have found that loneliness is a stressor and positively associated with perceived stress. Loneliness usually demonstrates the lack of social and intimate relationships which shrinks the ways to copy stress (27). As a result, it is easy to suffer psychological problems such as anxiety and depression.

Our results also found that resilience was an important mediator between the relationship of loneliness on anxiety and depression among Chinese elderly migrants. The mediating effects of resilience are 7.6 and 10.8%, respectively. This finding agrees with Kumpfer's resilience framework, where resilience can mediate the relationship between adversity and its outcomes (37). In this study, loneliness is regarded as an adversity, with anxiety and depression as its negative outcomes. As mentioned above, loneliness can limit individuals' recourses to seek help when they suffer adversities, which are a manifestation of resilience. However, resilience is helpful to increase the belief to overcome elderly migrants' negative outcomes (62). Thus, the relationships between loneliness, resilience, anxiety and depression among Chinese elderly migrants are clear. Loneliness can not only directly influence anxiety and depression, but also through the mediating effect of resilience. In addition, the comparisons of the mediating effects of perceived stress and resilience showed that the effects of perceived stress were significantly stronger than resilience both in the relationship between loneliness and anxiety and in the relationship between loneliness and depression. In short, perceived stress and resilience play crucial roles in explaining the relationships of loneliness on anxiety and depression among Chinese elderly migrants. For elderly migrants who feel lonely, they are more likely to suffer anxiety and depression. According to the findings of this paper, the interventions that combine components of reducing perceived stress or increasing resilience are important to improve their anxiety and depression. The community can organize some activities and lectures to provide a platform for elderly migrants to get out of their house and communicate and interact with others. And adult children should communicate more with their elderly parents with the experience of migration to identify and help them cope with adversities in a timely manner.

## Limitation

Firstly, the cross-sectional research design makes it difficult to reveal causal relationships between the key variables. Secondly, the information collected through participant self-reports may affect the reliability of our results, although we have assessed the reliability of the used scales and gained a positive result. Thirdly, the project was not originally designed to measure the effects of loneliness on psychological distress, so some potential control variables were not measured. For example, the age at which older migrants move may have an impact on our results, as those who move at age 50 and older may have different experiences than those who move at age 60 and older. Lastly, all participants were recruited from Nanjing, Jiangsu Province, and may not apply to other regions with different geographic and cultural backgrounds. In future studies, conduct a longitudinal study In future studies, a longitudinal study design is needed to clarify the causal relationship between loneliness and psychological disorders. In addition, we will try to collect samples of elderly migrants from different regions of China and fully consider the possible influencing factors to fill the gaps in the current study.

## Conclusion

This study was an important extension of the literature on elderly migrants, not only confirming the important role of loneliness in anxiety and depressive symptoms, but also finding the crucial mediating roles of perceived stress and resilience. The present findings showed that loneliness was positively associated with anxiety and depressive symptoms among Chinese elderly migrants. Loneliness can not only directly affect anxiety and depressive symptoms, but also affect them by increasing perceived stress or decreasing resilience. Loneliness, anxiety and depression are prevalent in elderly people, and all of them can pose serious threats to elderly people' health and wellbeing. The findings of this study suggest that loneliness puts elderly migrants at risk for anxiety and depressive symptoms. But we can mitigate the negative effects of loneliness by reducing the level of perceived stress or increasing the level of psychological resilience (especially by reducing perceived stress).

## Data availability statement

The raw data supporting the conclusions of this article will be made available by the authors, without undue reservation.

## Ethics statement

The studies involving human participants were reviewed and approved by the Ethics Committee of Nanjing Medical University. Written informed consent for participation was not required for this study in accordance with the national legislation and the institutional requirements.

## Author contributions

HW: conceptualization, methodology, formal analysis, and writing—original draft. YH: formal analysis and writing—original draft. LZ, MY, and RD: investigation and data curation. JY: conceptualization, supervision, and writing—review and editing. All authors have approved the final manuscript.

## Funding

This work was supported by the National Social Science Foundation of China (number: 18BRK026).

## Conflict of interest

The authors declare that the research was conducted in the absence of any commercial or financial relationships that could be construed as a potential conflict of interest.

## Publisher's note

All claims expressed in this article are solely those of the authors and do not necessarily represent those of their affiliated organizations, or those of the publisher, the editors and the reviewers. Any product that may be evaluated in this article, or claim that may be made by its manufacturer, is not guaranteed or endorsed by the publisher.

## References

[1] National Health Commission of China. Report on China's Migrant Population Development. Beijing: China Population Publishing House (2018).

[2] Donato KM Caron L Hamilton E. Migration and mental health in mexico: domestic migrants, return US migrants, and non-migrants. Front Psychiatry. (2020) 10:970. 10.3389/fpsyt.2019.00970 32116812 PMC7008711

[3] Zheng X Zhang Y Chen Y Fang X. Internal migration experience and depressive symptoms among middle-aged and older adults: evidence from China. Int J Environ Res Public Health. (2022) 19:303. 10.3390/ijerph19010303 35010562 PMC8744975

[4] Chan ASW Ho JMC Li JSF Tam HL Tang PMK. Impacts of COVID-19 pandemic on psychological well-being of older chronic kidney disease patients. Front Med. (2021) 8:666973. 10.3389/fmed.2021.666973 34124096 PMC8187602

[5] Djukanovic I Carlsson J Arestedt K. Is the Hospital Anxiety and Depression Scale (HADS). a valid measure in a general population 65–80 years old? A psychometric evaluation study. Health Qual Life Outcomes. (2017) 15:193. 10.1186/s12955-017-0759-9 28978356 PMC5628437

[6] Lee SL Pearce E Ajnakina O Johnson S Lewis G Mann F. The association between loneliness and depressive symptoms among adults aged 50 years and older: a 12-year population-based cohort study. Lancet Psychiatry. (2021) 8:48–57. 10.1016/S2215-0366(20)30383-7 33181096 PMC8009277

[7] Zhao W Zhang Y Liu X Yue J Hou L Xia X. Comorbid depressive and anxiety symptoms and frailty among older adults: Findings from the West China health and aging trend study. J Affect Disord. (2020) 277:970–6. 10.1016/j.jad.2020.08.070 33065841

[8] Aziz R Steffens DC. What are the causes of late-life depression? Psychiatr Clin N Am. (2013) 36:497. 10.1016/j.psc.2013.08.001 24229653 PMC4084923

[9] Chang Y-C Yao G Hu SC Wang J-D. Depression affects the scores of all facets of the WHOQOL-BREF and may mediate the effects of physical disability among community-dwelling older adults. PLoS ONE. (2015) 10:128356. 10.1371/journal.pone.0128356 26010571 PMC4444229

[10] Dong L Freedman VA Mendes de Leon CF. The association of comorbid depression and anxiety symptoms with disability onset in older adults. Psychosom Med. (2020) 82:158–64. 10.1097/PSY.0000000000000763 31688675 PMC7007837

[11] Lenze EJ Wetherell JL. A lifespan view of anxiety disorders. Dialogues Clin Neurosci. (2011) 13:381–99. 10.31887/DCNS.2011.13.4/elenze 22275845 PMC3263387

[12] Patel RS Wardle K Parikh RJ. Loneliness: the present and the future. Age Ageing. (2019) 48:476–7. 10.1093/ageing/afz026 30927406

[13] Mao ZH Zhao XD. The effects of social connections on self-rated physical and mental health among internal migrant and local adolescents in Shanghai, China. BMC Public Health. (2012) 12:97. 10.1186/1471-2458-12-97 22299776 PMC3305514

[14] Nørredam M. Migration and health: exploring the role of migrant status through register-based studies. Dan Med J. (2015) 62:B5068. 25872539

[15] Spagnoli L Flahault A Ferrara P. Migrant health burden: where do we stand? Int J Environ Res Public Health. (2020) 17:3004. 10.3390/ijerph17093004 32357449 PMC7246684

[16] Hawkley LC Cacioppo JT. Loneliness matters: a theoretical and empirical review of consequences and mechanisms. Ann Behav Med. (2010) 40:218–27. 10.1007/s12160-010-9210-8 20652462 PMC3874845

[17] Cacioppo JT. The growing problem of loneliness. Lancet. (2018) 391:426–426. 10.1016/S0140-6736(18)30142-9 29407030 PMC6530780

[18] Yang K Victor CR. The prevalence of and risk factors for loneliness among older people in China. Ageing Soc. (2008) 28:305–27. 10.1017/S0144686X0700684823714357

[19] Leigh-Hunt N Bagguley D Bash K Turner V Turnbull S Valtorta N. An overview of systematic reviews on the public health consequences of social isolation and loneliness. Public Health. (2017) 152:157–71. 10.1016/j.puhe.2017.07.035 28915435

[20] Creese B Khan Z Henley W O'Dwyer S Corbett A Da Silva MV. Loneliness, physical activity, and mental health during COVID-19: a longitudinal analysis of depression and anxiety in adults over the age of 50 between 2015 and 2020. Int Psychoger. (2021) 33:505–14. 10.1017/S1041610220004135 33327988 PMC7985900

[21] Cacioppo JT Hawkley LC Thisted RA. Perceived social isolation makes me sad: 5-year cross-lagged analyses of loneliness and depressive symptomatology in the Chicago Health, Aging, and Social Relations Study. Psychol Aging. (2010) 25:453–63. 10.1037/a0017216 20545429 PMC2922929

[22] O'Connor DB Thayer JF Vedhara K. Stress and health: a review of psychobiological processes. Annu Rev Psychol. (2021) 72:663–88. 10.1146/annurev-psych-062520-122331 32886587

[23] Cohen S Kamarck T Mermelstein R. A global measure of perceived stress. J Health Soc Behav. (1983) 24:385–96. 10.2307/2136404 6668417

[24] Liu Z Liu R Zhang Y Zhang R Liang L Wang Y. Association between perceived stress and depression among medical students during the outbreak of COVID-19: the mediating role of insomnia. J Affect Disord. (2021) 292:89–94. 10.1016/j.jad.2021.05.028 34107425 PMC8595067

[25] Burke TJ Segrin C. Bonded or stuck? Effects of personal and constraint commitment on loneliness and stress. Person Ind Differ. (2014) 64:101–6. 10.1016/j.paid.2014.02.027

[26] Christiansen J Larsen FB Lasgaard M. Do stress, health behavior, and sleep mediate the association between loneliness and adverse health conditions among older people? Soc Sci Med. (2016) 152:80–6. 10.1016/j.socscimed.2016.01.020 26849687

[27] Cacioppo JT Hawkley LC Berntson GG. The anatomy of loneliness. Curr Direct Psychol Sci. (2003) 12:71–4. 10.1111/1467-8721.01232

[28] Huang L-J Du W-T Liu Y-C Guo L-N Zhang J-J Qin M-M. Loneliness, stress, and depressive symptoms among the chinese rural empty nest elderly: a moderated mediation analysis. Issues Ment Health Nurs. (2019) 40:73–8. 10.1080/01612840.2018.1437856 30633625

[29] Li S Ye X Wang L Li Y Yang H Liu X. The effect of perceived stress and social support between loneliness and mental health among the solitary elderly (In Chinese). Chongqing Med. (2018) 47:4044–7, 4052.

[30] Zheng S Men R Fan Z Fu L Wan T. Loneliness on depression among elderly hypertensive patients: the mediating role of perceived stress (In Chinese). Chin J Gerontol. (2021) 41:5768–71.

[31] Connor KM Davidson JRT. Development of a new resilience scale: The Connor-Davidson Resilience scale (CD-RISC). Depress Anxiety. (2003) 18:76–82. 10.1002/da.10113 12964174

[32] Foster JR. Successful coping, adaptation and resilience in the elderly: an interpretation of epidemiologic data. Psychiatr Q. (1997) 68:189–219. 10.1023/A:1025432106406 9237317

[33] Bauer H Emeny RT Baumert J Ladwig KH. Resilience moderates the association between chronic pain and depressive symptoms in the elderly. Eur J Pain. (2016) 20:1253–65. 10.1002/ejp.850 26914727

[34] Liu JJW Ein N Gervasio J Battaion M Reed M Vickers K. Comprehensive meta-analysis of resilience interventions. Clin Psychol Rev. (2020) 82. 10.1016/j.cpr.2020.101919 33045528

[35] MacLeod S Musich S Hawkins K Alsgaard K Wicker ER. The impact of resilience among older adults. Am J Geriatr Psychiatry. (2016) 24:S157. 10.1016/j.jagp.2016.02.029 27055911

[36] McKinley CE Boel-Studt S Renner LM Figley CR. Risk and protective factors for symptoms of depression and anxiety among american indians: understanding the roles of resilience and trauma. Psychol Trauma Theory Res Pract Policy. (2021) 13:16–25. 10.1037/tra0000950 32940525 PMC7814695

[37] Kumpfer KL. Factors and Processes Contributing to Resilience, Resilience and Development. Springer (2002). p. 179–224.

[38] Zhao X Zhang D Wu M Yang Y Xie H Li Y. Loneliness and depression symptoms among the elderly in nursing homes: a moderated mediation model of resilience and social support. Psychiatry Res. (2018) 268:143–51. 10.1016/j.psychres.2018.07.011 30025285

[39] Zhong X Wu D Nie X Xia J Li M Lei F. Parenting style, resilience, and mental health of community-dwelling elderly adults in China. BMC Geriatr. (2016) 16:135. 10.1186/s12877-016-0308-0 27391781 PMC4938943

[40] Kong L-N Zhang N Yuan C Yu Z-Y Yuan W Zhang G-L. Relationship of social support and health-related quality of life among migrant older adults: The mediating role of psychological resilience. Geriatr Nurs. (2021) 42:1–7. 10.1016/j.gerinurse.2020.10.019 33197701

[41] Hughes ME Waite LJ Hawkley LC Cacioppo JT. A short scale for measuring loneliness in large surveys: results from two population-based studies. Res Aging. (2004) 26:655–72. 10.1177/0164027504268574 18504506 PMC2394670

[42] Li H Wang C. The relationships among structural social support, functional social support, and loneliness in older adults: analysis of regional differences based on a multigroup structural equation model. Front Psychol. (2021) 12:732173. 10.3389/fpsyg.2021.732173 34650489 PMC8507854

[43] Liu T Lu S Leung DK Sze LC Kwok WW Tang JY. Adapting the UCLA 3-item loneliness scale for community-based depressive symptoms screening interview among older Chinese: a cross-sectional study. BMJ Open. (2020) 10:e041921. 10.1136/bmjopen-2020-041921 33303463 PMC7733209

[44] Yang TZ Huang HT. An epidemiological study on stress among urban residents in social transition period (In Chinese). Chin J Epidemiology. (2003) 24:760–4. 14521764

[45] Tan T Leung CW. Associations between perceived stress and BMI and waist circumference in Chinese adults: data from the 2015 China Health and Nutrition Survey. Public Health Nutr. (2021) 24:4965–74. 10.1017/S1368980020005054 33308370 PMC11082812

[46] Campbell-Sills L Stein MB. Psychometric analysis and refinement of the Connor-davidson Resilience Scale (CD-RISC): validation of a 10-item measure of resilience. J Trauma Stress. (2007) 20:1019–28. 10.1002/jts.20271 18157881

[47] Qiu C Shao D Yao Y Zhao Y Zang X. Self-management and psychological resilience moderate the relationships between symptoms and health-related quality of life among patients with hypertension in China. Qual Life Res. (2019) 28:2585–95. 10.1007/s11136-019-02191-z 31049824

[48] Zhang D Wang R Zhao X Zhang J Jia J Su Y. Role of resilience and social support in the relationship between loneliness and suicidal ideation among Chinese nursing home residents. Aging Ment Health. (2021) 25:1262–72. 10.1080/13607863.2020.1786798 32602736

[49] Zigmond AS Snaith RP. The hospital anxiety and depression scale. Acta Psychiatr Scand. (1983) 67:361–70. 10.1111/j.1600-0447.1983.tb09716.x 6880820

[50] Bjelland I Dahl AA Haug TT Neckelmann D. The validity of the Hospital Anxiety and Depression Scale. An updated literature review. J Psychosomat Res. (2002) 52:69–77. 10.1016/S0022-3999(01)00296-3 11832252

[51] Arean PA Ayalon L. Assessment and treatment of depressed older adults in primary care. Clin Psychol Sci Pract. (2005) 12:321–35. 10.1093/clipsy.bpi034

[52] Liu ZW Yu Y Hu M Liu HM Zhou L Xiao SY. PHQ-9 and PHQ-2 for screening depression in Chinese rural elderly. PLoS ONE. (2016) 11:e0151042. 10.1371/journal.pone.0151042 26978264 PMC4792401

[53] Xu WQ Lin LH Ding KR Ke YF Huang JH Hou CL. The role of depression and anxiety in the relationship between poor sleep quality and subjective cognitive decline in Chinese elderly: exploring parallel, serial, and moderated mediation. J Affect Disord. (2021) 294:464–71. 10.1016/j.jad.2021.07.063 34325166

[54] Hayes AF. Introduction to Mediation, Moderation, and Conditional Process Analysis Third Edition: A Regression-Based Approach. New York, NY: Guilford Publications (2022).

[55] Wang J Mann F Lloyd-Evans B Ma R Johnson S. Associations between loneliness and perceived social support and outcomes of mental health problems: a systematic review. BMC Psychiatry. (2018) 18:156. 10.1186/s12888-018-1736-5 29843662 PMC5975705

[56] Weiss R. Loneliness: The Experience of Emotional and Social Isolation. Cambridge, MA: MIT Press (1975).

[57] Drageset J Espehaug B Kirkevold M. The impact of depression and sense of coherence on emotional and social loneliness among nursing home residents without cognitive impairment - a questionnaire survey. J Clin Nurs. (2012) 21:965–74. 10.1111/j.1365-2702.2011.03932.x 22250600

[58] Peerenboom L Collard RM Naarding P Comijs HC. The association between depression and emotional and social loneliness in older persons and the influence of social support, cognitive functioning and personality: a cross-sectional study. J Affect Disord. (2015) 182:26–31. 10.1016/j.jad.2015.04.033 25965692

[59] Liu G Li S Kong F. Association between sense of belonging and loneliness among the migrant elderly following children in Jinan, Shandong Province, China: the moderating effect of migration pattern. Int J Environ Res Public Health. (2022) 19:4396. 10.3390/ijerph19074396 35410076 PMC8998737

[60] van Tilburg TG Fokkema T. Stronger feelings of loneliness among Moroccan and Turkish older adults in the Netherlands: in search for an explanation. Eur J Ageing. (2021) 18:311–22. 10.1007/s10433-020-00562-x 34483796 PMC8377113

[61] Segrin C Passalacqua SA. Functions of loneliness, social support, health behaviors, and stress in association with poor health. Health Commun. (2010) 25:312–22. 10.1080/10410231003773334 20512713

[62] Liang D Teng M Xu D. Impact of perceived social support on depression in Chinese rural-to-urban migrants: the mediating effects of loneliness and resilience. J Community Psychol. (2019) 47:1603–13. 10.1002/jcop.22215 31332801

